# Longitudinal associations between exclusive, dual and polytobacco use and respiratory illness among youth

**DOI:** 10.1186/s12889-024-19582-8

**Published:** 2024-08-08

**Authors:** Luis Zavala-Arciniega, Steven Cook, Jana L. Hirschtick, Yanmei Xie, Richa Mukerjee, Douglas Arenberg, Geoffrey D. Barnes, David T. Levy, Rafael Meza, Nancy L. Fleischer

**Affiliations:** 1 Department of Epidemiology, University of Michigan School of Public Health, 1415 Washington Heights, Ann Arbor, MI 48109 USA; 2 Division of Pulmonary & Critical Care Medicine, University of Michigan Medical School, Ann Arbor, MI USA; 3 Division of Cardiovascular Medicine, Department of Internal Medicine, University of Michigan, Ann Arbor, USA; 4 Lombardi Comprehensive Cancer Center, Georgetown University, Washington, DC USA; 5 Department of Integrative Oncology, BC Cancer Research Institute, Vancouver, BC Canada

**Keywords:** Polytobacco use, Respiratory illness, Epidemiology

## Abstract

**Background:**

The health consequences of polytobacco use are not well understood. We evaluated prospective associations between exclusive, dual, and polytobacco use and diagnosed bronchitis, pneumonia, or chronic cough among US youth.

**Methods:**

Data came from Waves 1–5 of the Population Assessment of Tobacco and Health Study. We categorized time-varying past 30-day tobacco use into seven categories: (1) non-current use; exclusive use of 2) cigarettes, 3) e-cigarettes, and 4) other combustible products (OC; pipes, hookah, and cigars); dual use of 5) e-cigarettes + cigarettes or e-cigarettes + OC, and 6) cigarettes + OC; and 7) polyuse of all three products. The outcome was parent-reported diagnosis of bronchitis, pneumonia, or chronic cough among youth. We conducted weighted multilevel Poisson models (person *n* = 17,517, 43,290 observations) to examine the longitudinal exposure-outcome relationship, adjusting for covariates: sex, age, race and ethnicity, parental education, body mass index, secondhand smoke exposure, and household use of combustible products.

**Results:**

Compared to nonuse, exclusive cigarette use (Risk Ratio (RR) = 1.83, 95% CI 1.25–2.68), exclusive e-cigarette use (RR = 1.53, 95% CI 1.08–2.15), combustible product + e-cigarette dual use (RR = 1.90, 95% CI 1.18–3.04), cigarettes + OC dual use (RR = 1.96, 95% CI 1.11–3.48), and polytobacco use (RR = 3.06 95% CI 1.67–5.63) were associated with a higher risk of bronchitis, pneumonia, or chronic cough. In additional analyses, we found that the risk ratio for polytobacco use was higher compared to exclusive e-cigarette use (RR 2.01 CI 95% 1.02–3.95), but not higher compared to exclusive cigarette use (RR 1.67 CI 95% 0.85–3.28).

**Conclusion:**

We found that exclusive, dual, and poly tobacco use were all associated with higher risk of bronchitis, pneumonia, or chronic cough compared to non-current use.

**Supplementary Information:**

The online version contains supplementary material available at 10.1186/s12889-024-19582-8.

## Introduction

Tobacco use is an important cause of morbidity among youth [1]. For example, combustible tobacco product use has been identified as a risk factor for acute respiratory diseases [2, 3]. However, little is known about the respiratory health consequences of exclusive, dual (use of two products), and polyuse (three or more products) of tobacco products, which is important due to the continued introduction of new tobacco products [4, 5]. Exclusive, dual, and poly tobacco use patterns are evolving for youth. A recent study using data from 2014 to 2019 found that, among youth, exclusive e-cigarette use increased (from 3.2 to 12.8%), while exclusive cigarette use and dual/polyuse without e-cigarettes decreased [6]. Given the rapidly changing tobacco product landscape, it is critical to understand the relationship between exclusive, dual, and polytobacco use and respiratory health outcomes among youth and young adults to better understand if the combination of using multiple tobacco products result in greater respiratory disease risk.

Previous studies have found that cigarette use is associated with increased risk of acute respiratory health outcomes among youth and young adults [7, 8]. Results from PATH studies reported that dual use combustible tobacco products (i.e., cigarette and cigars) was associated with a higher incidence of asthma at follow-up compared to non-use of tobacco products [9]. In addition, clinical and population studies have found that current cigarette use (vs. never cigarette use) was associated with bronchitis and acute pneumonia among youth and young adults [7, 10–12]. Clinical studies and systematic reviews found that e-cigarette use was associated with adverse respiratory health outcomes [13–20]. Additionally, one longitudinal study in California among high schools students found that current e-cigarette use (vs. nonuse) was associated with a higher risk of respiratory symptoms [21]. Moreover, systematic review articles that include cross-sectional and longitudinal studies found an association between e-cigarette use and asthma [16–18]. However, two nationally representative longitudinal studies published in 2023 found that exclusive e-cigarette use and dual use of cigarettes and e-cigarettes at baseline were not associated with higher asthma incidence among youth at follow-up [9, 22]. While the asthma association has been prospectively evaluated at the national level, there is a need to evaluate the link between exclusive, dual, and polytobacco use with short-term respiratory outcomes such as acute bronchitis or pneumonia. One recent study from our team found that the exclusive cigarette use, exclusive e-cigarette use, and dual use of cigarettes and e-cigarettes was associated with bronchitis, pneumonia, and chronic cough [23]. However, no studies have evaluated the link between polytobacco use and the risk of bronchitis, pneumonia, and chronic cough. We aim to fill this gap by studying this association using data from six waves of the PATH survey, a nationally representative longitudinal study, in order to provide additional insights about the short-term health consequences of polyuse of tobacco products among youth. We hypothesize that the risk of bronchitis, pneumonia, or chronic cough will be higher among those who cigarettes, e-cigarettes, and other combustible products together.

## Methods

**Fig. 1:**
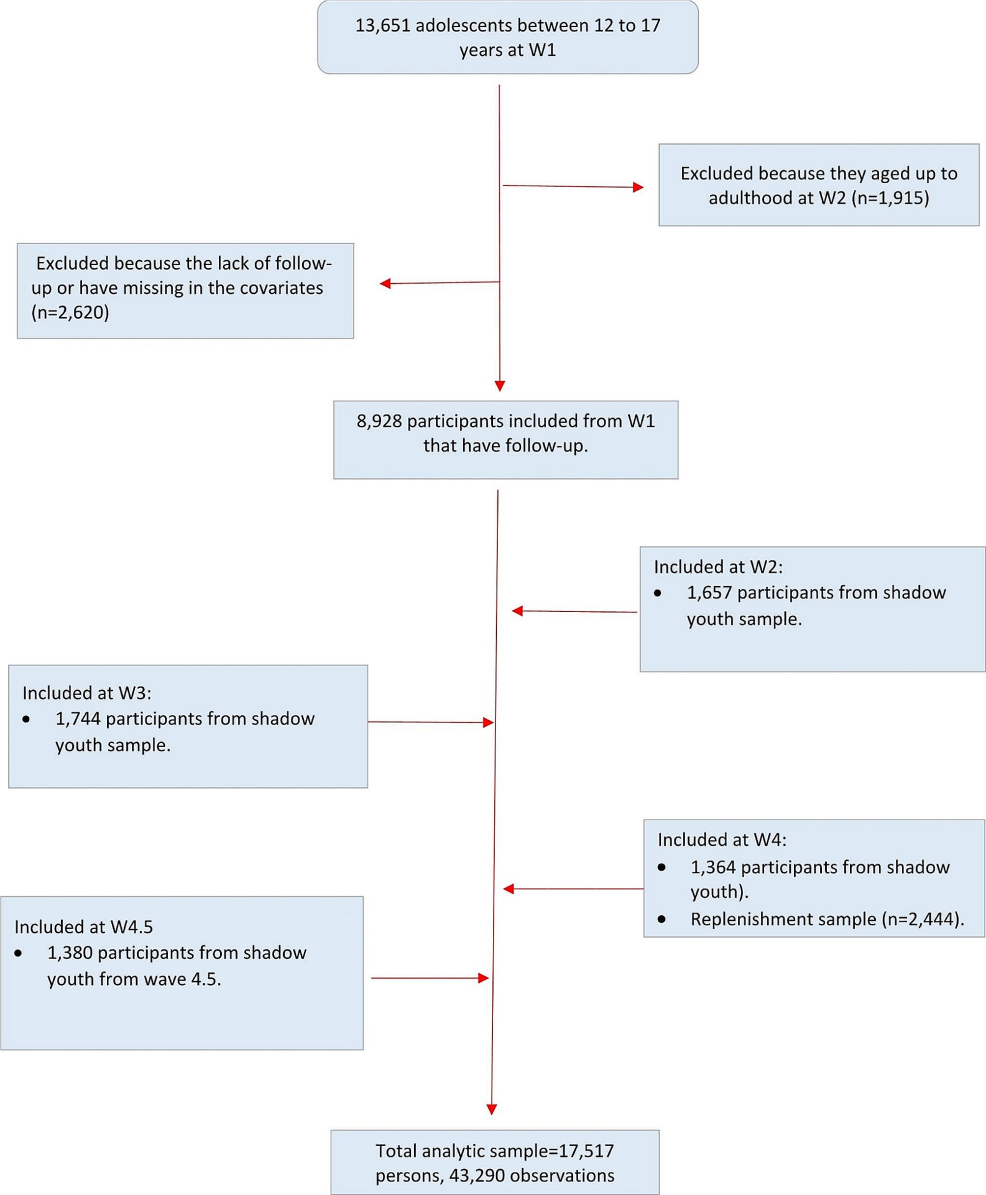
Analytic sample flowchart

We used restricted youth data from Waves 1 to 5 of the PATH Study, including Wave 4.5. The analytic sample consisted of youth between 12 and 17 years who completed at least one follow-up survey. We also included participants who aged up into the youth sample between waves 2 and 4.5 (shadow youth) and youth from the replenishment sample in Wave 4. We consider baseline data for each respondent as data from their first interview, which could occur between Waves 1 and 4.5 (See Fig. 1). Data were collected using audio computer self-interviews (ACASI) in English and Spanish in the following periods: Wave 1 from September 2013 to December 2014; Wave 2 from October 2014 to October 2015; Wave 3 from October 2015 to October 2016; Wave 4 from December 2016 to January 2018; Wave 4.5 from December 2017 to November 2018; and Wave 5 from December 2018 to November 2019. A detailed description of the methodology of the PATH study has been published elsewhere [24, 25]. Given the use of de-identified datasets, the University of Michigan Institutional Review Board deemed this project not regulated as human subject’s research.

Figure titles:

### Parent-reported bronchitis, pneumonia, or chronic cough incidence

We evaluated parent-reported bronchitis, pneumonia, or chronic cough among youth participants. Parents of the youth participants were queried, *“In the past 12 months*,* has (Child’s first name) been told by a doctor*,* nurse*,* or other health professional that (he/she) has bronchitis*,* pneumonia*,* or chronic cough?”* The study outcome was measured at each wave, starting in wave 2, and could occur more than once.

### Exclusive, dual, and poly tobacco use as time-dependent exposure variable

For the exposure, we categorized time-varying past 30-day tobacco use into 7 mutually exclusive categories: (1) non-current/never tobacco use; exclusive use of (2) cigarettes, (3) electronic nicotine delivery systems (e-cigarettes), and (4) other combustibles (OC; pipes, hookah, and cigars); dual use of (5) e-cigarettes + combustible tobacco (cigarettes or OC), and (6) cigarettes + OC; and (7) polyuse of all three tobacco product groups. The e-cigarettes + combustible tobacco dual use category was created as a combined e-cigarettes + cigarettes and e-cigarettes + OC category due to (a) small sample sizes for e-cigarettes + cigarettes and e-cigarettes + OC, and (b) the hypothesized similar health effect for e-cigarettes plus any combustible product. Current tobacco product use was defined as smoking cigarettes/cigars or using e-cigarettes in the past 30 days. We lagged the exposure variable by one wave (t-1) to ensure that the tobacco variable exposure preceded the bronchitis, pneumonia, or chronic cough outcome (i.e., if exposure was measured at Wave 1, the outcome was measured at Wave 2).

### Covariates

We included sex (female, male); race and ethnicity (Non-Hispanic (NH) White, NH Black, Hispanic, and Another Race/Ethnicity (including multiracial)); parental education (less than high school, high school, some college, and bachelor’s degree or higher); body mass index (normal/underweight, overweight, obese); and household use of combustibles products (no, yes) as baseline covariates at the time of respondent’s first interview. Secondhand smoke exposure to tobacco combustible products was measured in number of hours exposed in the past 7 days. We also included as adjustment variables ever cannabis use (yes/no), and asthma (yes/no). Age was included as a categorical variable (12–14 and 15–17 years).

### Analysis

First, we created a person-period data set containing multiple responses per participant (*n* = 17,517; 43,290 observations). We then calculated descriptive statistics for the sociodemographic characteristics and risk factor distributions at baseline for our analytic sample. We also calculated the time-varying prevalence of the exclusive, dual, and polyuse tobacco exposure variable by wave and the cigarette smoking intensity pattern by wave. Finally, we conducted unadjusted and adjusted weighted, multilevel Poisson models to examine the longitudinal exposure-outcome relationship across five different periods (Waves 1–2, Waves 2–3, Waves 3–4, Waves 4-4.5, and Waves 4.5-5). Multilevel models were used because the outcome could occur more than once, and these models adjusted for the lack of independence of the repeated observations. Adjusted models included the covariates described above.

All estimates adjusted for the sample design by recalibrating the PATH weights into two-level weights to accommodate the longitudinal hierarchy of the data [26]. Briefly, Level-1 weights were the conditional wave-specific weights that were scaled, and their sum is equal to the number of data points available for each participant in the study. Level-2 weights were the baseline weights. In other words, Level-2 weights were the cross-sectional weights in which individuals began in the study (i.e., Wave 1 for most participants, Waves 2-4.5 for aged-up youth, and Wave 4 for youth recruited for the replenishment sample) [26]. We conducted the statistical analysis using Stata 18.1. We also conducted additional analyses to compare risk across the different products. We examined the risk of e-cigarettes compared to cigarettes, (2) dual use compared to cigarettes, (3) polyuse compared to cigarettes, and (4) polyuse compared to e-cigarettes (See Appendix 1a and 1b). In addition, to ensure that covariates included in the main analysis were not biasing the results, we conducted sensitivity analyses that excluded participants who had ever used cannabis at baseline (Appendix 2) and models without the asthma covariate (Appendix 3).

## Results

Table 1 shows the baseline sociodemographic characteristics and covariate distribution for participants in our analytic sample at their baseline year (*n* = 17,517). The baseline year corresponds to the wave that the participant entered the study. Just over half the participants were male (51.5%). More than half of respondents (53%) were NH White, 13% were NH Black, 24% were Hispanic, and 10% were from another race/ethnicity. About 40% of participants reported having a parent with a bachelor’s degree or higher. Approximately 27% of the participants reported that someone in their household used tobacco products A total of 7.4% of the sample reported bronchitis, pneumonia, or chronic cough across the study period. Among respondents who reported the outcome (*N* = 1,309), about 20% (*n* = 264) reported the outcome more than once over the study period.

**Table 1:** Baseline sociodemographic characteristics and smoking behavior, population assessment of tobacco & health study (Wave 1, 2013, 2014) among youth (*n* = 17,517)

	%	95% CI	*n*
**Sex**			
Male	51.5	[50.7, 52.2]	9098
Female	48.5	[47.7, 49.3]	8416
Age*			
12–14 years	60.5	[59.4,61.6]	5437
15–17 years	39.5	[38.4,40.6]	3525
**Race/ethnicity**			
NH White	53.0	[40.2,53.8]	8390
NH Black	12.9	[12.4,13.5]	2280
Hispanic	24.1	[23.4,24.7]	5160
Another Race/Ethnicity	10.0	[9.5,10.5]	1678
**Parental education**			
Less than high school	13.0	[12.5,13.5]	2686
High school	20.7	[20.1,21.4]	3853
Some college	27.0	[26.2,27.7]	4730
Bachelor’s degree or higher	39.2	[38.5,40.5]	6248
***Baseline risk factors***			
**Second hand smoke exposure (10 h)**	0.3	[0.3,0.3]	17,517
**Ever marijuana use**			
No	**93.6**	[92.2,94.0]	16,289
Yes	**6.4**	[6.0,8.8]	1228
**Ever asthma diagnosis**			
No	**81.9**	[81.3,82.5]	14,288
Yes	**18.1**	[17.5,18.7]	3229
**Household use of combustible products**			
No	73.5	[72.7,74.1]	12,667
Yes	26.5	[25.9,27.3]	4850
**BMI - obesity**			
Normal/underweight	64.8	[64,0,65.4]	11,064
Overweight	18.3	[17.7,18.9]	3290
Obese	17.0	[16.3,17.6]	3163
**Bronchitis**, Pneumonia or Chronic cough episodes**			
None	92.6	[92.2,93.0]	16,208
One	5.9	[5.5,6.2]	1045
Two or more	1.5	[1.3,1.7]	264

*Table 1 only includes the distribution of age at W1. Age was included as time-variant in the models

**Bronchitis, pneumonia and Chronic cough episodes over the study period. From W2 to W5

**Fig. 2:**
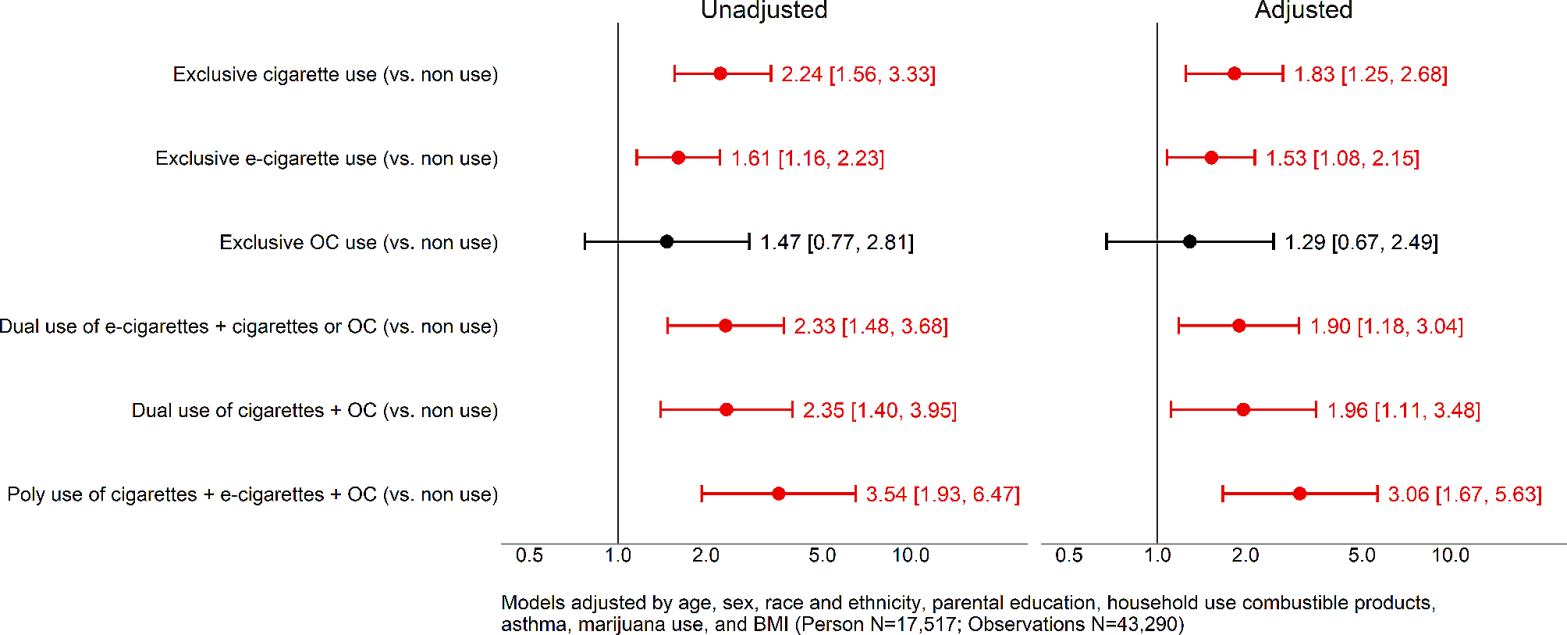
Risk of bronchitis, pneumonia and chronic cough among youth

Table 2 describes the changes in the tobacco exposure variable across waves. The prevalence of exclusive use of cigarettes and OC decreased from Wave 1 to Wave 5 (from 1.5 to 0.8%), while exclusive e-cigarette use increased (from 1.1 to 3.8%). Dual use of e-cigarettes with cigarettes or OC did not change from Wave 1 to Wave 5, but cigarette + OC use decreased. Polyuse of tobacco products remained at about 0.3% over the study period. Table 3 shows the cigarette smoking intensity pattern by wave. Cigarette smoking intensity was not statistically different among the exclusive cigarette use, dual use, and polytobacco use categories over time. The results from the multilevel Poisson regression models can be found in Fig. 2; Table 4. There were 43,290 observations in the models corresponding to *n* = 17,517 respondents. In the adjusted models, compared to non-current use of tobacco products, exclusive cigarette use (RiskRatio [RR] = 1.83, 95% CI 1.25–2.68) and exclusive e-cigarette use (RR = 1.53, 95% CI 1.08–2.15) were associated with a higher risk of diagnosed bronchitis, pneumonia, or chronic cough. The risk of diagnosed bronchitis, pneumonia, or chronic cough was also higher for dual use of exclusive e-cigarette use + combustible tobacco (IRR = 1.90, 95% CI 1.18–3.04), dual use of cigarettes + OC (RR = 1.96, 95% CI 1.11–3.48), and polytobacco use (RR = 3.06 95% CI 1.67–5.63), compared to non-current use of tobacco products. The only tobacco use category that was not statistically different from non-current use was exclusive OC (RR = 1.29, 95% CI 0.67–2.49). We also found that polytobacco use was associated with a higher risk of respiratory illness when compared to exclusive e-cigarette use (RR 2.01 CI 95% 1.02–3.95), but not when compared to exclusive cigarette use (RR 1.67 CI 95% 0.85–3.28.). In addition, when we compared use categories to exclusive cigarette use as the reference group, dual use of cigarettes and e-cigarettes (RR 1.04, 95% CI 0.59–1.82) and exclusive e-cigarette use (RR 0.83, 95% CI 0.51–1.37) were not statistically different (See Appendix 1a and 1b). Findings from the sensitivity analyses (see Appendix 2 and 3), were consistent with the main findings in terms of the direction of association and statistical significance.

**Table 2:** Time-dependent tobacco exposure variable by wave (*n* = 43,290 observations)

	Wave 1 (*n* = 8928)		
*Time varying poly tobacco use variable*	%	95% CI	*n*
Non use	95.1	[94.6, 95.5]	8485
Exclusive cigarette use	1.5	[1.2, 1.8]	132
Exclusive e-cigarette use	1.1	[0.9, 1.3]	91
Exclusive other combustible use	0.7	[0.5, 0.9]	66
Dual use of cigarettes + ENDS/ Dual use of ENDS + OC	0.7	[0.6, 0.9]	64
Dual use of cigarettes + other combustibles	0.5	[0.4, 0.7]	48
Poly use of cigarettes + ENDS + OC	0.4	[0.3, 0.6]	43
	**Wave 2 (** ***n*** ** = 8,271)**		
Non use	95.0	[94.5, 95.5]	7855
Exclusive cigarette use	1.3	[1.1, 1.6]	113
Exclusive e-cigarette use	1.4	[1.2, 1.7]	114
Exclusive other combustible use	0.8	[0.6, 1.0]	68
Dual use of cigarettes + ENDS/ Dual use of ENDS + OC	0.7	[0.5, 0.9]	59
Dual use of cigarettes + other combustibles	0.5	[0.3, 0.6]	40
Poly use of cigarettes + ENDS + OC	0.3	[0.2, 0.4]	22
	**Wave 3 (** ***n*** ** = 7,825)**		
Non use	95.6	[95.1, 96.1]	7469
Exclusive cigarette use	0.9	[0.7, 1.2]	70
Exclusive e-cigarette use	1.9	[1.6, 2.2]	151
Exclusive other combustible use	0.4	[0.3, 0.6]	38
Dual use of cigarettes + ENDS/ Dual use of ENDS + OC	0.6	[0.5, 0.9]	54
Dual use of cigarettes + other combustibles	0.3	[0.2, 0.5]	27
Poly use of cigarettes + ENDS + OC	0.2	[0.1, 0.3]	16
	**Wave 4 (** ***n*** ** = 9,614)**		
Non use	95.6	[95.1, 96.0]	9192
Exclusive cigarette use	1.0	[0.8, 1.2]	99
Exclusive e-cigarette use	2.0	[1.7, 2.4]	178
Exclusive other combustible use	0.4	[0.3, 0.6]	41
Dual use of cigarettes + ENDS/ Dual use of ENDS + OC	0.6	[0.5, 0.8]	64
Dual use of cigarettes + other combustibles	0.2	[0.1, 0.3]	19
Poly use of cigarettes + ENDS + OC	0.2	[0.1, 0.3]	21
	**Wave 4.5 (** ***n*** ** = 8,652)**		
	%	95% CI	*n*
Non use	93.8	[93.3, 94.4]	8108
Exclusive cigarette use	0.8	[0.6, 1.0]	75
Exclusive e-cigarette use	3.8	[3.4, 4.3]	328
Exclusive other combustible use	0.3	[0.2, 0.4]	26
Dual use of cigarettes + ENDS/ Dual use of ENDS + OC	0.8	[0.6, 1.0]	72
Dual use of cigarettes + other combustibles	0.2	[0.1, 0.3]	18
Poly use of cigarettes + ENDS + OC	0.3	[0.2, 0.4]	25

**Table 3:** Time-dependent smoking intensity variable (cigarettes smoked per day = CPD) by wave

	Wave 1		
	Mean cigarettes per day (CPD)	95% CI	*n*
Exclusive cigarette use	1.73	[1.15, 2.32]	132
Dual use of cigarettes + ENDS or ENDS + OC	1.85	[0.87, 2.82]	64
Dual use of cigarettes + OC	1.62	[0.87, 2.38]	48
Polyuse of cigarettes + ENDS + OC	2.38	[1.12, 3.64]	43
	**Wave 2**		
Exclusive cigarette use	1.86	[1.04, 2.69]	111
Dual use of cigarettes + ENDS or ENDS + OC	1.62	[0.76, 2.49]	59
Dual use of cigarettes + OC	3.71	[2.30, 5.12]	40
Polyuse of cigarettes + ENDS + OC	0.96	[0.30, 1.62]	22
	**Wave 3**		
Exclusive cigarette use	1.61	[0.86, 2.36]	70
Dual use of cigarettes + ENDS or ENDS + OC	2.19	[0.84, 3.54]	54
Dual use of cigarettes + OC	2.49	[1.12, 3.86]	27
Polyuse of cigarettes + ENDS + OC	2.43	[0.88, 3.99]	16
	**Wave 4**		
Exclusive cigarette use	1.20	[0.68, 1.72]	99
Dual use of cigarettes + ENDS or ENDS + OC	0.52	[0.21, 0.82]	64
Dual use of cigarettes + OC	2.68	[0.08, 5.28]	19
Polyuse of cigarettes + ENDS + OC	1.32	[0.69, 1.95]	21
	**Wave 4.5**		
Exclusive cigarette use	1.11	[0.53, 1.69]	75
Dual use of cigarettes + ENDS or ENDS + OC	0.74	[0.20, 1.29]	72
Dual use of cigarettes + OC	3.30	[0.83, 5.76]	19
Polyuse of cigarettes + ENDS + OC	1.60	[0.24, 2.96]	25

**Table 4:** Multilevel models predicting risk of of bronchitis, pneumonia and chronic cough among youth respondents (12–17), population assessment of tobacco & health study (Wave 1–5, 2013-19) (*n* = 17,517 persons, 43,290 observations)

	Unadjusted				Adjusted			
	Risk Ratio (RR)	95% CI		*p*-value	RR	95% CI		*p*-value
***Time varying poly tobacco use variable***								
Non use	REF		REF		REF		REF	
Exclusive cigarette use	**2.24**	**1.56**	**3.22**	**0.000**	**1.83**	**1.25**	**2.68**	**0.002**
Exclusive e-cigarette use	**1.61**	**1.16**	**2.23**	**0.005**	**1.53**	**1.08**	**2.15**	**0.015**
Exclusive other combustible use	1.47	0.77	2.81	0.248	1.29	0.67	2.49	0.435
Dual use of e-cigarettes + cigarettes or OC	**2.33**	**1.48**	**3.68**	**0.000**	**1.90**	**1.18**	**3.04**	**0.008**
Dual use of cigarettes + OC	**2.35**	**1.40**	**3.95**	**0.001**	**1.96**	**1.11**	**3.48**	**0.021**
Poly use of cigarettes + ENDS + OC	**3.54**	**1.93**	**6.47**	**0.000**	**3.06**	**1.67**	**5.62**	**0.000**
**Age**								
12–14 years	REF		REF		REF		REF	
15–17 years	1.12	1.00	1.26	0.054	1.04	0.92	1.17	0.540
**Sex**								
Male	REF		REF		REF		REF	
Female	1.08	0.95	1.23	0.254	1.20	1.05	1.38	0.007
**Race/ethnicity**								
NH White	REF		REF		REF		REF	
NH Black	0.76	0.61	0.95	0.016	0.68	0.54	0.84	0.000
Hispanic	0.69	0.59	0.81	0.000	0.66	0.56	0.79	0.000
NH Other	0.75	0.59	0.95	0.017	0.71	0.56	0.89	0.007
**Parental education**								
Less than high school	REF		REF		REF		REF	
High school	1.00	0.79	1.25	0.969	0.95	0.75	1.19	0.651
Some college	1.25	1.01	1.56	0.043	1.19	0.95	1.49	0.121
Bachelor’s degree or higher	1.05	0.85	1.30	0.652	1.08	0.86	1.35	0.511
**Second hand smoke exposure (10 h)**	1.10	1.06	1.13	0.000	1.04	1.01	1.08	0.021
**Household use of combustible products**								
No	REF		REF		REF		REF	
Yes	1.33	1.15	1.53	0.000	1.09	0.94	1.27	0.257
**Eve marijuana use**								
No	REF				REF			
Yes	1.57	1.22	2.02	0.000	1.18	0.89	1.58	0.255
**BMI - obesity**								
Normal/underweigth	REF		REF		REF		REF	
Overweight	1.29	1.08	1.54	0.005	1.28	1.07	1.53	0.007
Obese	1.75	1.49	2.06	0.000	1.65	1.40	1.95	0.000
**Asthma**								
Yes	REF				REF			
No	2.99	2.6	3.44	0.000	2.99	2.6	3.44	0.00

## Discussion

Using data from a large and nationally representative longitudinal sample, we found associations between exclusive, dual, and polyuse of tobacco products and the risk of acute bronchitis, pneumonia, or chronic cough among youth. Our results shows that the use of exclusive cigarette use, exclusive e-cigarette use, dual use of e-cigarette use with combustible tobacco, dual use of cigarettes and OC, and polyuse of cigarettes, e-cigarette, and OC were all associated with higher risk of acute bronchitis, pneumonia, or chronic cough compared to non-use of tobacco products. We also found that polytobacco use was associated with a higher risk of bronchitis, pneumonia, and chronic cough compared to exclusive e-cigarette use.

The current dynamic landscape of the tobacco market has led to increased exclusive e-cigarette use and decreased cigarette use and dual use with cigarettes among youth during the years of our study. However, polytobacco use remained stable during the study period. In this context of availability of multiple tobacco products, we found that adolescents using cigarettes, e-cigarettes, or cigars exclusively or concurrently were at higher risk of developing short-term respiratory outcomes than adolescents who did not use tobacco. Moreover, our findings suggest that polyuse of tobacco products results in greater risk of bronchitis, pneumonia, or chronic cough. Therefore, policymakers should reinforce measures that restrict access to all tobacco products, including e-cigarette use, for adolescents to reduce their disease risk.

Our finding that exclusive e-cigarette use and e-cigarette use with combustible tobacco use (dual and poly) were both associated with a higher risk of acute bronchitis, pneumonia, or chronic cough suggests that e-cigarette use among youth affects acute respiratory health. This finding is generally consistent with findings from clinical population studies, which have found that e-cigarette use increases the risk of acute respiratory infections [27–30]. It is possible that e-cigarette use toxicants could directly damage lung function, which could exacerbate respiratory infections [16, 31–34]. However, bronchitis and pneumonia are both acute respiratory infections and it is also possible that adolescents are transmitting these infections by sharing e-cigarette devices with friends, as research shows is common [35]. Sharing e-cigarette devices potentially could lead to an increase in respiratory infections through the exchange of saliva. To better understand the mechanisms at play, future national surveys on tobacco use would benefit from incorporating questions about the sharing behaviors of e-cigarettes and other tobacco products among adolescents.

Consistent with our finding that exclusive cigarette use was associated with acute bronchitis, pneumonia, or chronic cough, previous studies have found that individual combustible tobacco use was associated with bronchitis and pneumonia [10, 11]. By examining dual and polytobacco use, we were further able to demonstrate that the use of two or more tobacco products is associated with bronchitis, pneumonia, and chronic cough. Of note, cigarette smoking intensity was not statistically different among adolescents who used two or more tobacco products, compared to those who used cigarettes exclusively (Table 3). Therefore, our finding suggests that using two or more tobacco products may further increase the risk of adverse acute respiratory health outcomes.

In contrast, we found that exclusive OC tobacco product use was not statistically associated with an increased risk of incident bronchitis, pneumonia, and chronic cough, but OC use was associated with an increased incident risk when used in combination with other tobacco products. The lack of a statistically significant association of exclusive OC use with the respiratory outcomes could be explained by the smaller sample size for the exclusive OC use category, even though the RR was above 1. Future research is warranted to examine the independent risk of each product (i.e., hookah, cigars, and pipes) in the OC use category.

This study has several limitations. First, parents were asked about bronchitis, chronic cough, and pneumonia as part of a single question in the PATH survey, so that it was not possible to separately examine these respiratory outcomes. Future longitudinal studies incorporating specific questions for each respiratory health outcome are warranted. Second, diagnosis of the respiratory outcomes was self-reported by the parents, which might introduce information bias. For example, we found that minoritized racial and ethnic groups had lower risk of a self-reported diagnosis of bronchitis, pneumonia, or chronic cough than NH White adolescents, which could be due to having lower access to health services [36]. Third, we cannot rule out residual confounding in our analyses. For example, the PATH study assessed urbanicity of residence only at Wave 1, so we were unable to adjust for it given the inclusion of the youth shadow samples (i.e., youth who began participating in the study in Wave 2 or after). Also, we did not adjust for neighborhood-level contextual variables because geographic variables are not available in the PATH study below the state level. Moreover, we did not adjust for household income because of high levels of missing values, which are most likely not randomly distributed. To overcome this limitation, we used parental educational attainment as measure of socioeconomic status. Previous studies have used parental educational attainment as a proxy for socioeconomic status [37, 38]. Fourth, the outcome survey question was only assessed for youth but not adults, limiting our ability to follow participants when they aged up into the adult sample. Fifth, although we included cannabis use as covariate, in the PATH youth survey the cannabis question measured ‘ever use of marijuana’ at baseline for each participant and we were not able to examine cannabis use as a time-varying covariate. Moreover, we were not able to differentiate between cannabis smoking and cannabis vaping. This is an important task for future research. Despite these limitations, this study is important because it provides new evidence of the relationship between specific tobacco products used alone or in combination and acute respiratory health among youth.

## Conclusion

In the context of the rapidly changing tobacco use patterns among youth, we found that, compared to non-current use, the exclusive use of cigarettes, the exclusive use of e-cigarettes, and the use of two or more tobacco products were associated with incident bronchitis, pneumonia, and chronic cough.

### Electronic supplementary material

Below is the link to the electronic supplementary material.

Supplementary Material 1

## Data Availability

The dataset generated and analyzed in the current study used restricted use data from the Population Assessment of Tobacco and Health (PATH) study. Details on how to access restricted use data from the PATH Study is described in the PATH Study Restricted Use Files User Guide: 10.3886/ICPSR36231.v37. For questions related to the study results or queries from this study please contact the correspondent author [Luis Zavala-Arciniega/lzavalaa@umich.edu].
